# Databases, big data and artificial intelligence: what healthcare professionals need to know about them

**DOI:** 10.1590/1516-3180.2022.140611082022

**Published:** 2022-09-23

**Authors:** Leticia Leone Lauricella, Paulo Manuel Pêgo-Fernandes

**Affiliations:** I MD, PhD. Attending Physician, Thoracic Surgery Service, Instituto do Câncer do Estado de São Paulo, Hospital das Clinicas HCFMUSP, Faculdade de Medicina, Universidade de Sao Paulo; II MD, PhD. Full Professor, Thoracic Surgery Program, Instituto do Coração (InCor), Hospital das Clinicas HCFMUSP, Faculdade de Medicina, Universidade de Sao Paulo, Sao Paulo, SP, BR; Director, Scientific Department, Associação Paulista de Medicina (APM), São Paulo (SP), Brazil.; Associação Paulista de Medicina, Scientific Department, São Paulo, SP, Brazil

*“Information is the oil of the 21st century, and analytics is the combustion engine”* said Peter Sondergaard, senior vice president of Gartner Research. The more information we have, the more likely we are to find correlations that are not obvious to the eye and that can completely change the way we think or act. We are living in a reality where tons of data from several different sources are routinely collected, often without we realizing it. Every aspect of our lives, such as social activities, consumption patterns, Internet searches, geographic movements on global positioning systems, and health issues, are somehow being transformed in data. The large volume of new data being generated is straining the capacity of institutions to manage and of researchers to make use of them, a situation has been termed the “Data Deluge”.^1^

One of the most notable areas where data analytics is causing makeovers is in healthcare. Apart from traditional medical research, there are numerous other sources with the potential to contribute to big data. Some examples include hospital records, patient's medical records, results of medical examinations, and lots of new computing devices (known as the “internet of things”), which are embedded in everyday objects and can collect real-time body-signals ubiquitously. Through the analysis of large amounts of data, diseases can be diagnosed earlier, thus improving the prognosis of serious diseases. Medical costs can be reduced, epidemics can be predicted, diseases can be prevented and quality of life can be improved. At the individual level, we are increasingly moving into the era of personalized medicine, where the information of a given patient, especially his/her genetic data, will be analyzed and processed for the establishment of a specific and personalized treatment.

Since the early 2000s, when the term *big data* came into use, the way data are collected and analyzed has completely changed. The famous 3 “Vs” of big data refers to volume, velocity and variety.^2^ Although, other people have added several other Vs to this definition such as veracity, value, visualization, and variability, in the end, we are talking about data with sizes that exceed the capacity of traditional software to process within an acceptable time and *value*. Another good explanation for big data is that it “involves all the data collections endowed with a sufficient “size” and lack of definition (having been assembled with no *a priori* hypothesis or specific research task) to be considered as still largely unspoiled territories from where to derive new insights in the form of unforeseen regularities”.^3^

The European Commission developed the following definition for “big data in Health”: it refers to large routinely or automatically collected datasets, which are electronically captured and stored. It is reusable in the sense of multipurpose data and comprises the fusion and connection of existing databases for the purpose of improving health and health system performance. It does not refer to data collected for a specific study.” ^4^

In the medical field, with the advancements of radiomics for example, a method that extracts a large number of features from medical images using data-characterization algorithms, millions of data can be extracted automatically through software developed for this purpose, feeding large data repositories that will be used to predict outcomes, dispense new tests or optimize diagnostic investigation, reducing costs and improving the treatment of numerous diseases. ^5^

Imagine the following situation: you, as a healthcare professional, have a patient with a lung nodule found in a thorax computed tomography scan. With a single chest tomography, through the analysis of radiomics and artificial intelligence, you can determine if the nodule is malignant and what the anatomopathological subtype is, sparing the patient from the risks of a biopsy. The analysis of lung parenchyma, heart area, coronary calcifications, muscle mass and subcutaneous tissue will provide information about lung and cardiac function, sarcopenia and malnutrition, allowing a precise prediction of postoperative complications and again saving the patient from being submitted to numerous preoperative examinations. Additionally, a detailed analysis of the anatomical structures of this lung, with the recognition of possible anatomical variations, will allow a better surgical planning, in such a way that the surgeon will know in advance the difficulties he will encounter during the surgery.

Therefore, this is the future and it will probably be a reality in approximately 10 years. However, to get there we need large amounts of data; not only data automatically extracted from imaging examinations or medical devices, but also clinical data. Because that is how systems learn, that is how artificial intelligence and machine learning work. It means that constructing and feeding clinical databases are an essential step to get to the future of big data.

However, building and maintaining a clinical database is not an easy task. Using the example of lung cancer again, there are large international multicentric databases ^6,7,8^ that still rely on the human effort for data imputation, despite the fact that technology already exists for extracting information directly from electronic health records (EHRs). This fact is a major constraint for large-scale quality data collection, especially in countries like Brazil where there are no national or governmental initiatives for the development of medical registries, and the vast majority of health professionals are not familiar with data collection outside clinical research.

There are many challenges associated with big data in healthcare. Even in the United States, where the adoption of federally tested and certified EHR programs in the healthcare sector is nearly complete, the existence of different programs, with different clinical terminologies, technical specifications, and functional capabilities has led to difficulties in the interoperability and sharing of data.^2^ Furthermore, most EHR systems contain lots of unstructured data, making it more complex to extract useful information for big data. Drawing a parallel between the United States and Brazil, Brazil are a long way from this problem yet, as many health services are either still implementing their EHR systems or simply still rely on manual health records.

Furthermore, having an EHR system available in a given health service does not mean that data will actually be extracted to contribute to a clinical database. It probably will not be because, in addition to all the technological difficulties listed above, there are other barriers related to regulatory laws on accessing and sharing personal information, such as *the General Data Protection Law (“LGPD”*) in Brazil, the *General Data Protection Regulation* (“GDPR”) in Europe, and the *Health Insurance Portability and Accountability Act* (HIPAA) in United States. To buttress this point, solutions still need to be found or agreed for many issues, especially in Brazil, where the LGPD is relatively recent. First of all, who owns patients' information - hospitals, researchers, or patients themselves? Assuming that the information belongs to the patient, how can this contribute to the development of big data and ultimately to the improvement of the patient's own health? How can sensitive data related to health issues be collected in large volumes with patients' consent and privacy protection?

Owing to all this, despite all the advances in technology, a lot of health data are still being “lost” daily, even in large referral services, simply because there is no initiative for the prospective collection of such data. Addressing this problem requires, first of all, recognizing the importance of data collection for the development of science on a national scale. We urgently need initiatives for the development of national medical specialties databases and cancer registries. We need to know the dimensions of our own data to compare our numbers to international benchmarks instead of just consuming international data and trying extrapolate them to our locality.
